# Statistical Approaches for the Analysis of Dependency Among Neurons Under Noise

**DOI:** 10.3390/e22040387

**Published:** 2020-03-28

**Authors:** Deniz Gençağa, Sevgi Şengül Ayan, Hajar Farnoudkia, Serdar Okuyucu

**Affiliations:** 1 Department of Electrical and Electronics Engineering, Antalya Bilim University, 07190 Antalya, Turkey; serdar.okuyucu@antalya.edu.tr; 2 Department of Industrial Engineering, Antalya Bilim University, 07190 Antalya, Turkey; sevgi.sengul@antalya.edu.tr; 3 Department of Statistics, Middle East Technical University, 06800 Ankara, Turkey; hajar.farnoudkia@metu.edu.tr

**Keywords:** transfer entropy, mutual information, information theory, copulas, Hodgkin–Huxley model

## Abstract

Neuronal noise is a major factor affecting the communication between coupled neurons. In this work, we propose a statistical toolset to infer the coupling between two neurons under noise. We estimate these statistical dependencies from data which are generated by a coupled Hodgkin–Huxley (HH) model with additive noise. To infer the coupling using observation data, we employ copulas and information-theoretic quantities, such as the mutual information (MI) and the transfer entropy (TE). Copulas and MI between two variables are symmetric quantities, whereas TE is asymmetric. We demonstrate the performances of copulas and MI as functions of different noise levels and show that they are effective in the identification of the interactions due to coupling and noise. Moreover, we analyze the inference of TE values between neurons as a function of noise and conclude that TE is an effective tool for finding out the direction of coupling between neurons under the effects of noise.

## 1. Introduction

The Hodgkin–Huxley (HH) model is one of the most famous mathematical models describing the behavior of action potentials in neurons using a set of nonlinear differential equations [1]. Action potentials are also known as the spikes. Each spike is generated as a result of a potential with a value exceeding a specific threshold value [2,3,4].

HH model is a nonlinear dynamical system and the governing equations are deterministic. However, neuronal networks are influenced by random electrical fluctuations, which are also known as the neuronal noise. Examples include thermal, 1/f, ionic conductance, ionic shot and synaptic noise [5]. Among these, synaptic noise has larger amplitudes that can cause the neural system dynamics to change. In any noisy case, the model has a stochastic nature due to the random components. Therefore, in the literature, the HH model is extended as a stochastic system [6,7,8]. Stochastic resonance [9] is a typical example of noise effecting a system, wherein action potentials can be triggered by the additive contributions of the noise amplitude, leading to a value higher than the threshold. An action potential can also be suppressed by the negative effect of a random noise sample. Therefore, the noise is not merely a nuisance factor and it can change the meaning of the “neuronal code” via changes in spike trains. In the literature, neuronal noise is examined in many aspects. Horikawa examines the effects of membrane current noise on spike propagation using the stochastic HH model [8]. In [6], Goldwyn and Shea-Brown present a review of channel noise models, while White et al. elaborate on the quantification of this noise in addition to the models of the populations of stochastic ion channels [10]. Ermentrout et al. state the noisy nature of the brain and emphasize the constructive role of noise [11] by showing the noise-induced synchronization of neurons. Among many contributions on this topic, Faisal et al. [12] present a review on the potential benefits of noise; Lee et al. study first and second order phase transitions in neuronal networks stimulated by shot noise [13]; and Lindner et al. provide an extensive review on the behavior of theoretical models of excitable systems driven by Gaussian white noise [14], wherein they utilize models such as the leaky integrate-and-fire and the FitzHugh-Nagumo.

Relationships between neurons and noise are also studied by the utilization of novel statistical signal processing methods and information-theoretic learning techniques [15]. Cohen and Kohn review works on correlation analysis and investigate the factors affecting the estimation and measurement of correlations [16]. Yamada et al. analyze the dependency between three and then between *N* neurons by estimating information-theoretical quantities, such as the mutual information [17,18]. Wibral et al. present a review on the directional information measures, such as time-lagged mutual information (MI), directed information, cross entropy and transfer entropy (TE) [19]. Among these measures, TE is widely preferred in the literature to detect the information flow, and it has many variants, such as the normalized permutation transfer entropy (NPTE) [20], extended TE [21] and trial-shuffle-based TE [22], which are utilized to identify the connectivity between neurons.

In our work, we propose a statistical toolset to analyze the relationships of neurons under noise. We utilize information theory and copulas to better understand the effects of noise on the interactions between neurons and we test the performances of these approaches. We demonstrate the performances of the information-theoretical quantities as functions of different noise levels. The results are supported by copulas, which are widely employed in the literature to describe the dependencies between random variables [23]. In its most general form, interactions between neurons involve multivariate non-Gaussian probability distributions due to non-linearities involved in the system dynamics. We can learn the joint probability density functions (pdfs) of a pair of statistically dependent variables from observation data in a non-parametric way using copulas. Copulas are utilized to map marginal distributions of variables into their joint distribution [23]. Different types of copulas have been proposed in the literature, such as Gaussian, Clayton and Gumbel [23]. One of the methods for the decision of the optimal copula is based on the Vuong and Clarke tests [24,25,26]. Copulas are also ideal for determining the tail dependencies. 

As we outline above, our statistical toolset includes the estimation of informational theoretical quantities. Based on the estimations of MI values between the action potential data of two neurons, we observed a decaying dependency until a certain level of noise, which was followed by an increasing dependency. This behavior was also verified by the results of copula analysis. Although we can infer the statistical dependency between neurons, MI and copulas are not enough to detect the direction of the interaction between them. Thus, we conclude our dependency analysis by estimating the TE values between neurons as a function of noise levels. The analysis demonstrates that unlike the MI and copulas, TE keeps decreasing despite the increasing noise level. As a result, we can conclude that TE is effective at inferring the direction of interactions at low noise levels. Moreover, MI and copulas are effective for inferring the magnitude of the dependency under different noise levels, and they are instrumental to better understanding the effects of noise and coupling coefficient as two different factors acting on the dependencies between neurons.

The organization of this paper is as follows: In the next section, we present brief background material on neuroscience, information theory and copulas. After that, we focus on our approach for the analysis of the problem and provide the results before the conclusions.

## 2. Materials and Methods

In this section, we present how we model the interactions between neurons under noise. The background material on the statistical approaches to inferring these relationships follow, and the section on the steps of data generation is given next.

### 2.1. The Hodgkin–Huxley Model 

In this work, we use coupled Hodgkin–Huxley (HH) equations under noise to test the performances of the information-theoretical quantities and copulas while inferring the interactions between the modeled neurons. HH is a mathematical model describing the action potential generation, and it is one of the major breakthroughs in computational neuroscience [1]. Following the seminal work of Sir Hodgkin and Sir Huxley in 1952, HH type models were defined for many different electrically-excitable cells, such as cardiomyocytes [2], pancreatic beta cells [3] and hormone secretion [4] due to the observations of cell membranes behaving much like electrical circuits. The basic circuit elements are the phospholipid bilayer of the cell, which behaves like a capacitor that accumulates ionic charge while the electrical potential across the membrane changes. Moreover, resistors in a circuit are analogous to the ionic permeabilities of the membrane, and the electrochemical driving forces are analogous to batteries driving the ionic currents. Na^+^, K^+^, Ca^2+^ and Cl^−^ ions are responsible for almost all the electrical actions in the body. Thus, the electrical behavior of cells is based upon the transfer and storage of ions, and K^+^ and Na^+^ ions are mainly responsible for the HH system.

The HH model relates the membrane potential *V* to the conservation of electric charge, as shown below:

(1) $$C_{m}\frac{dV}{dt}=I_{ion}+I_{app}\;,$$

where $C_{m}$ is the membrane capacitance, $I_{app}$ is the applied current and $I_{ion}$ represents the sum of individual ionic currents which is modeled according to the Ohm’s law as follows:

(2) $$I_{ion}=-g_{K}\left(V-V_{K}\right)-g_{Na}\left(V-V_{Na}\right)-g_{L}\left(V-V_{L}\right).$$

Here $g_{K}$, $g_{Na}$ and $g_{L}$ are conductances; $V_{Na}$, $V_{K}$, $V_{L}$ are the reversal potentials associated with the currents. The details of this model can be found in Appendix A.

### 2.2. Information Theory Quantities

Information theory sets the rules for the quantification, storage and flow of information in many areas, such as statistics, physics, engineering, computer science and neuroscience. The fundamental information theoretic quantity is known as the Shannon entropy and it is defined to be the average uncertainty for finding the system at a particular state “*v*” out of a possible set of states “*V*”, where *p*(*v*) denotes the probability of that state. It is also used to quantify the amount of information needed to describe a dataset. For discrete variables, Shannon entropy is given by the following formula:

(3) $$H\left(V\right)=-\underset{v\in V}{\sum }p\left(v\right)\log\left(p\left(v\right)\right).$$

Mutual information (MI) is another fundamental information-theoretic quantity which is used to quantify the "amount of information" obtained about one random variable through observing the other random variable. Given two datasets denoted by *V*_1_ and *V*_2_, the MI can be written as follows:

(4) $$MI\left(V_{1},V_{2}\right)=\underset{v_{1}\in V}{\sum }\;\underset{v_{2}\in V}{\sum }p\left(v_{1},v_{2}\right)log\frac{p\left(v_{1},v_{2}\right)}{p\left(v_{1}\right)p\left(v_{2}\right)}.$$

The MI is a symmetric quantity and it can be rewritten as a sum and difference of Shannon entropies by

(5) $$MI\left(V_{1},V_{2}\right)=H\left(V_{1}\right)+H\left(V_{2}\right)-H\left(V_{1},V_{2}\right)\;,$$

where $H\left(V_{1},V_{2}\right)$ is the joint Shannon entropy. If there is a directional dependency between the variables, such as a cause and effect relationship, a symmetric measure cannot unveil this information from data. In the literature, TE was proposed to analyze the directional dependencies between two Markov processes [27]. To quantify the directional effect of a variable $V_{1}$ on $V_{2}$, TE is defined by the conditional distribution of $V_{2}$ depending on the past samples of both processes versus the conditional distribution of that variable depending only on its own past values [27]. Thus, the asymmetry of TE helps us detect the information flow in two directions. The TE definitions in both directions (between variables $V_{1}$ and $V_{2}$) are given by the following equations:

(6) $$\begin{array}{c} TE_{V_{1}V_{2}}=T(V_{2_{\left(i+1\right)}}|{V_{2}}_{\left(i\right)}^{\left(k\right)},{V_{1}}_{\left(i\right)}^{\left(l\right)})\\ \;=\underset{v_{2}{}_{\left(i+1\right)},v_{2}{}_{\left(i\right)}^{\left(k\right)},v_{1}{}_{\left(i\right)}^{\left(l\right)}}{\sum }p\left(v_{2}{}_{\left(i+1\right)},v_{2}{}_{\left(i\right)}^{\left(k\right)},v_{1}{}_{\left(i\right)}^{\left(l\right)}\right)\log_{2}\frac{p\left(v_{2}{}_{\left(i+1\right)}|v_{2}{}_{\left(i\right)}^{\left(k\right)},v_{1}{}_{\left(i\right)}^{\left(l\right)}\right)}{p\left(v_{2}{}_{\left(i+1\right)}|v_{2}{}_{\left(i\right)}^{\left(k\right)}\right)} \end{array}$$

(7) $$\begin{array}{c} TE_{V_{2}V_{1}}=T\left(V_{1}{}_{\left(i+1\right)}|V_{1}{}_{\left(i\right)}^{\left(k\right)},V_{2}{}_{\left(i\right)}^{\left(l\right)}\right)=\\ \sum_{v_{1}{}_{\left(i+1\right)},v_{1}{}_{\left(i\right)}^{\left(k\right)},v_{2}{}_{\left(i\right)}^{\left(l\right)}}p\left(v_{1}{}_{\left(i+1\right)},v_{1}{}_{\left(i\right)}^{\left(k\right)},v_{2}{}_{\left(i\right)}^{\left(l\right)}\right)\log_{2}\frac{p\left(v_{1}{}_{\left(i+1\right)}|v_{1}{}_{\left(i\right)}^{\left(k\right)},v_{2}{}_{\left(i\right)}^{\left(l\right)}\right)}{p\left(v_{1}{}_{\left(i+1\right)}|v_{1}{}_{\left(i\right)}^{\left(k\right)}\right)} \end{array}$$

where indices (*i* + 1) and (*i*) denote the current time and its preceeding instant, respectively. Above, ${v_{1}}_{\left(i\right)}^{\left(k\right)}=\left\{{v_{1}}_{\left(i\right)},\ldots ,{v_{1}}_{\left(i-k+1\right)}\right\}$ shows the vector including the value of $V_{1}$ at time instant (*i*) and its values at (*k* − 1) preceeding time instants. Similarly, ${v_{2}}_{\left(i\right)}^{\left(l\right)}=\left\{{v_{2}}_{\left(i\right)},\ldots ,{v_{2}}_{\left(i-l+1\right)}\right\}$ denotes the vector including the value of $V_{2}$ at time instant (*i*) and its values at (*l* − 1) preceeding time instants. Here, $V_{1}\;\mathrm{and}\;V_{2}$ denote *k*-th and *l*-th order Markov processes, respectively. In the literature, *k* and *l* are also known as the embedding dimensions. In our work, we choose *k* = *l* = 1, which means that one past value of each signals is taken into consideration during TE estimation. In this case, TE can be estimated by the marginal and joint Shannon entropies as $TE_{V_{2}V_{1}}=T\left({V_{1}}_{\left(i+1\right)}|{V_{1}}_{\left(i\right)},{V_{2}}_{\left(i\right)}\right)=H\left({V_{1}}_{\left(i\right)},{V_{2}}_{\left(i\right)}\right)-H\left({V_{1}}_{\left(i+1\right)},{V_{1}}_{\left(i\right)},{V_{2}}_{\left(i\right)}\right)+H\left({V_{1}}_{\left(i+1\right)},{V_{1}}_{\left(i\right)}\right)-H\left({V_{2}}_{\left(i\right)}\right)$. All these quantities involve estimation of probability distributions from observation data, without any prior information on the generation mechanism (in our case the HH equations). Despite the varieties in distribution estimation techniques in the literature, the whole procedure still suffers from adverse effects, such as the bias. Most common techniques in probability distribution estimation involve histograms, Parzen windows and adaptive methods [28,29,30,31,32]. Among them all, histogram estimation is widely used due to its computational simplicity. To rely on estimations from data, reporting the statistical significance of each estimate [33] constitutes an important part of the methods. In this work, we utilize the uniform count bin histogram-based method to estimate the distributions in (3), (4), (6) and (7), due to its computational simplicity [33]. In order to assess the statistical significance of the estimations, surrogate data testing is applied with a significance level of p=0.05. Next, we introduce copulas.

### 2.3. Copulas

A copula is a multivariate cumulative distribution function (cdf) for which the marginal probability distribution of each variable is continuous. If we apply the probability integral transform [23] to each component of a random vector, which is represented by $\left(V_{1},V_{2},\ldots ,V_{d}\right)$, we can express the uniformly distributed variables as shown below:

(8) $$\left(U_{1},U_{2},\ldots ,U_{d}\right)=\left(F_{1}\left(V_{1}\right),\ldots ,F_{d}\left(F_{d}\right)\right)$$

where $\left(U_{1},U_{2},\ldots ,U_{d}\right)$ are uniformly distributed variables between zero and one. These are obtained by the application of the cdf of each variable, represented by $F_{i}\left(V_{i}\right).$ The copula of $\left(U_{1},U_{2},\ldots ,U_{d}\right)$ is defined as the joint cdf of these uniformly distributed marginals by C(.) as shown below:

(9) $$C\left(u_{1},u_{2},\ldots ,u_{d}\right)=\mathrm{Pr}\left(U_{1}\leq u_{1},\ldots ,U_{d}\leq u_{d}\right)$$

Therefore, a *d*-dimensional copula is a joint cumulative distribution function of a *d*-dimensional random vector on the unit cube ${\left[0,1\right]}^{d}$ with uniform marginals, and it is represented by $C:{\left[0,1\right]}^{d}\to \left[0,1\right]$.

According to the Sklar’s theorem [23], any multivariate cumulative distribution function can be written in terms of a copula and cdfs as follows:

(10) $$H\left(V_{1},V_{2},\ldots ,V_{d}\right)=C\left(F_{1}\left(V_{1}\right),\ldots ,F_{d}\left(V_{d}\right)\right)$$

and the corresponding density function is given by the following equation:

(11) $$h\left(V_{1},V_{2},\ldots ,V_{d}\right)=c\left(F_{1}\left(V_{1}\right),\ldots ,F_{d}\left(V_{d}\right)\right)\cdot f_{1}\left(V_{1}\right)\ldots f_{d}\left(V_{d}\right).$$

Despite their convenient formulations, using multivariate copulas can be challenging in high dimensions [34]. Thus, in the literature, pairs of copulas are widely preferred. Paired copulas can be categorized in two main groups; namely, the elliptical and archimedean copulas [23]. The elliptical copulas are of the form of $C\left(u_{1},u_{2}\right)=F(F_{1}^{-1}\left(u_{1}\right),F_{2}^{-1}\left(u_{2}\right))$. The most famous elliptical copulas are bivariate Gaussian (one parameter) and bivariate Student’s *t* copulas (two parameters). In the literature, the cdf of a Gaussian distribution is generally denoted by ϕ [23]. The Gaussian copula and the Student’s *t* copula are both symmetric, and we can utilize Student’s *t* copula to model the tail dependencies between the variables, unlike the Gaussian copula. When two variables $V_{i}$ and $V_{j}$ are tail-dependent, a very large or very small value of $V_{i}$ is expected to be associated with a very large or very small value of $V_{j}$ and vice versa. The archimedean family contains more flexible copulas to model tail dependencies and non-symmetric cases. For the sake of completeness, Gaussian and archimedean copulas are briefly discussed below.

#### 2.3.1. Gaussian Copula

The Gaussian copula with parameter matrix ***R***, denoting the correlation matrix $R\in {\left[-1,1\right]}^{dxd}$ in *d* dimensions, is expressed as follows:

(12) $$C_{R}\left(u\right)=\phi_{R}\left(\phi^{-1}\left(u_{1}\right),\ldots ,\phi^{-1}\left(u_{d}\right)\right),$$

where $\phi^{-1}$ represents the inverse cdf of a standard univariate Gaussian, and $\phi_{R}$ denotes the joint cdf of a multivariate normal distribution with a zero mean and correlation matrix ***R***. Even though we cannot express this copula analytically, the density function of this distribution function can be given parametrically as follows:

(13) $$C_{R}\left(u\right)=\frac{1}{\sqrt[{\;}]{\det\left(R\right)}}\exp\left[-\frac{1}{2}{\left[\begin{array}{c} \phi^{-1}\left(u_{1}\right)\\ :\\ \phi^{-1}\left(u_{d}\right) \end{array}\right]}^{T}\left(R^{-1}-I\right)\left[\begin{array}{c} \phi^{-1}\left(u_{1}\right)\\ :\\ \phi^{-1}\left(u_{d}\right) \end{array}\right]\right],$$

where ***I*** is the identity matrix. 

#### 2.3.2. Archimedean Copulas

Unlike Gaussian copulas, most archimedean copulas can be written analytically. This copula family includes many flexible copulas catching the tail dependence (unlike Gaussians) and non-symmetric structure (unlike Gaussian and Student’s *t* copula). The whole list of archimedean copulas, some of which are Clayton, Frank, Gumbel and Joe, can be found in the literature [34]. For example, the Clayton copula is given in the following form:

(14) $$C\left(u_{1},u_{2}\right)=max\left({\left[u_{1}^{-\theta }+u_{2}^{-\theta }-1\right]}^{\frac{-1}{\theta }},\;0\right)$$

where θ denotes the copula parameter.

In one-parameter copulas, there is a one-to-one correspondence between the model parameter θ and the non-parametric correlation measure Kendall’s τ. For example, if the dependency is modeled by a Clayton copula; this is given as follows:

(15) $$\tau =\frac{\theta }{\theta +2}$$

In this work, we model statistical dependencies between two variables, namely, the action potentials of each neuron, using copulas, and we quantify the amounts of these dependencies by using Kendall’s τ values that are computed using the copula parameters. 

In conclusion, provided that the best copula is used to model the statistical dependency between the variables, the amount of this interaction can be provided by the estimate of τ. In the following sections, we will present how the best copula is chosen and how τ is calculated through the optimization of the copula parameter using observation data.

### 2.4. The Proposed Model and Statistical Analysis

In this work, we focus on the information-theoretic quantities and copulas to infer the interactions between neurons under noise effect. Our approach is based on four stages: (1) modeling, (2) dependency analysis by MI, (3) dependency analysis by copulas, (4) directional dependency analysis by TE. These are explained below.

#### 2.4.1. Modeling

In computational neuroscience, HH is one of the most common mathematical models used to generate action potentials. In this paper we focus on the system of two coupled HH neurons [35] with synaptic coupling from one neuron to another, to model the directional interaction between them. Such coupling corresponds to that of an electrical synapse, where the coupling is proportional to the difference between the pre-synaptic and postsynaptic membrane potentials. This type of coupling also refers to gap-junctions, as they allow the bi-directional transport of ions from one neuron to another [36]. An electrical synapse, which is independent of the conductance of ionic channels, is modelled using a coupling constant *k*. In order to model the effects of noise, we add independent, identically distributed Gaussian noise components to the HH equations. This two-neuron network involves fast sodium current $I_{i,Na}$, delayed rectifying potassium current $I_{i,K}$ and leak current $I_{i,L}\;\mathrm{for}\;i=1,2$. The differential equations for the rate of change of voltage for these neurons are given as follows:

(16) $$C_{m}\frac{dV_{1}}{dt}=I_{app}-I_{1,Na}-I_{1,K}-I_{1,L}+I_{noise},$$

(17) $$C_{m}\frac{dV_{2}}{dt}=-I_{2,Na}-I_{2,K}-I_{2,L}-k\left(V_{1}-V_{2}\right)+I_{noise},$$

where $I_{noise}$ denotes the noise component having a zero mean normal distribution with standard deviation σ, which is represented by N(0,σ) and $I_{app}$ represents the applied current shown in Equation (1). Here, $V_{1}$ and $V_{2}$ show the membrane voltages of the first and second neuron, respectively. Coupling between the two neurons is defined by the current $I=k\left(V_{2}-V_{1}\right)$, which is equal to the product of voltage difference and coupling strength k. When *k* is between 0 and 0.25, spiking activity occurs with unique stable limit cycle solution. After *k* = 0.25, the system turns back to stable steady state and spiking activity *disappears*. All other dynamics are the same as those described in Section 2.1. While the coupling constant changes the spike duration, it does not affect the spike amplitude.

#### 2.4.2. Dependency Analysis by MI

In the literature, correlation has been a fundamental tool for analyzing relationships between random variables for decades [37,38]. Although linear statistical dependencies can be inferred successfully, their performances degrade for nonlinear statistical relationships. For the latter case, information theory quantities, such as the MI, are much more effective [39,40]. Therefore, we propose to estimate the MI between the two neurons modeled by Equations (16) and (17). We analyze the performance of this quantity as a function of different noise levels. MI quantifies the amount of information obtained about one neuron through observing the other neuron. It is a symmetric quantity and it is zero for statistically independent variables, as can be seen from Equation (4).

#### 2.4.3. Dependency Analysis by Copulas

Copulas are multivariate cumulative distribution functions and they describe the dependencies between random variables. Copulas are applicable in situations where a correlation structure between two marginal distributions lacks a natural definition (for example, the covariance matrix of a multivariate Gaussian is defined). To infer the dependence between neurons from data, we suggest using bivariate copulas to analyze the relationships between $V_{1}\;\mathrm{and}\;V_{2}$ in the model. To analyze a relationship using a copula, the first stage is the proper selection of the copula type from data. Using empirical cumulative distribution functions which are obtained from data, we select the copula giving the highest score among 40 different types using the Vuong–Clarke test [24,25,26]. Having selected the copula type, the parameter *θ* is learned by maximum likelihood estimation over the observation data [34]. Finally, using the estimate of *θ*, we calculate Kendall’s τ between two neuron action potentials. In the end of this approach, we analyze the performances of copulas under different levels of noise.

#### 2.4.4. Directional Dependency Analysis by TE

Above, we emphasized that MI and copulas do not provide information about the direction of the coupling between the neurons. To analyze this, we propose to estimate the TE values between $V_{2}\;\mathrm{and}\;V_{1}$ in both directions in the case of no noise in Equations (16) and (17). After that, we include the noise components in Equations (16) and (17) and estimate TE between values between $V_{2}\;\mathrm{and}\;V_{1}$ as a function of increasing noise levels. Comparing these cases, we demonstrate the effects of noise on the information flow between the neurons. To estimate TE from data, we utilize the histogram approach [28,29,30,31] to infer the probability density functions in Equations (16) and (17). The significance of TE estimates are performed by surrogate data analysis [33] using a significance level of 0.05. Based on Equations (16) and (17), we know that the direction of the information flow must be from $V_{1}\;\mathrm{to}\;V_{2}$ under noiseless case. However, when noise is added, it is tedius to reach the same conclusion using the stochastic system dynamics governed by Equations (16) and (17) due to nonlinearities. Under different noise levels, we propose to estimate TE values from data, which is a non-parametric approach. 

## 3. Results

This section includes detailed information on the generation of data. In the following subsections, we present the results of our statistical approaches to infer the coupling between the neurons from data.

### 3.1. Data 

In this section, we analyze the interactions between two coupled neurons under noisy conditions using data generated by the model with equations given in Equations (16) and (17). The model is implemented in the XPPAUT software program [41] using the Euler method (dt = 0.1 ms), and the computer code is available as a Supplementary file. The related model parameters for 2-1 and 1-1 coupled neurons are provided in Table A1. Estimation of the information-theoretic quantities and copulas are executed in MATLAB 9.7 (R2019b) and R environments, respectively. For the simulations, parameters are taken to be as in Table 1.

**Figure 1:**
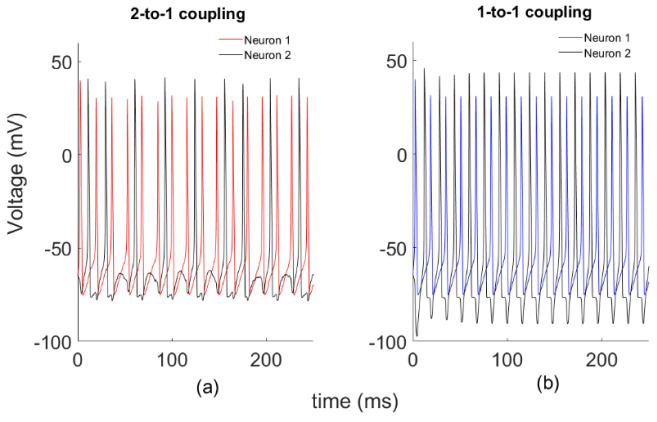
Sample spike patterns for two different spike configurations. (**a**) *k* = 0.1; (**b**) *k* = 0.25.

First, we study a system of two globally coupled HH models through a synapse by varying the coupling strength *k*, without noise’s effect. Phase dynamics of the systems for two different *k* coupling strengths are plotted in Figure 1. Here we define two different coupling patterns as shown below. In Figure 1a, Neuron 2 fires once after Neuron 1 fires twice, which we call 2-to-1 coupling with *k* = 0.1. For a larger coupling coefficient (*k* = 0.25), neurons show different synchronous firing patterns, as in Figure 1b. This time, each firing of Neuron 2 follows that of Neuron 1, which we call 1-to-1 coupling.

**Figure 2:**
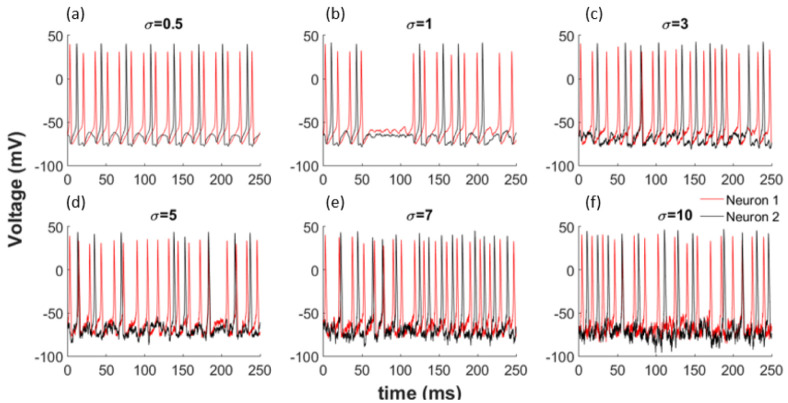
Noise effects the synchronization of the network. (**a**) σ = 0.5; (**b**) σ = 1; (**c**) σ = 3; (**d**) σ = 5; (**e**) σ = 7; (**f**) σ = 10.

To better understand the effects of noise on the network shown by Equations (16) and (17), we use zero mean Gaussian distributed random variables with standard deviation σ. When we incorporate this noise with different variances into our model, as illustrated in Equations (16) and (17), we observe a change in the synchronisation of the neurons. Additionally, the obvious patterns disappear totally for larger noise amounts as shown in Figure 2 for each coupled network. Noise can change the synchronization of neurons by inducing or deleting spikes in the network.

We analyzed the relationships between the neurons under noise by the implementation of our methods on batches of data over a duration of six seconds. To estimate each parameter of concern, such as the MI or TE, we run 10 Monte Carlo simulations using the noisy equations of Equations (16) and (17). We call a set of 10 different versions of data an ensemble, and denote each one of the 10 data sets an ensemble member. Having generated 10 data sets by Equations (16) and (17), we obtain 10 different values of parameter estimates for MI, TE and copula output, whose means and standard deviations are reported on graphs as functions of the additive noise levels.

### 3.2. Dependency Analysis by MI

**Figure 3:**
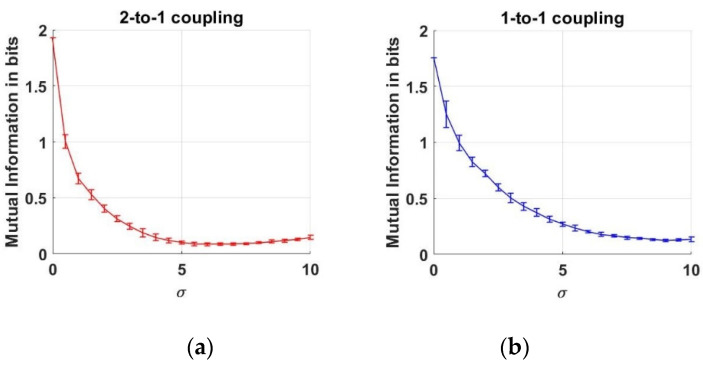
Mutual Information estimates between two neurons as a function of noise standard deviation for two coupling values. (**a**) 2-to-1 coupling case (*k* = 0.1); (**b**) 1-to-1 coupling case (*k* = 0.25).

Here, we estimate the MI between two neuron action potentials which are generated as described above. First, the joint and marginal pdfs shown in Equation (4) are estimated from data to estimate the MI between the action potentials. Throughout this work, we employ histograms to estimate the pdfs from data. In the literature, there are two main versions of histograms that are known as “uniform bin width” and “uniform count bin”. To estimate a pdf by “uniform bin width” histogram, the range between the minimum and maximum values of data is divided into a predetermined number of regions with constant sizes, which are known as the bin widths. The number of data falling into each bin determines the histogram values. To estimate a pdf by “uniform count bin” histogram, the bin sizes are arranged in such a way that the number of data counts in each bin are equal to each other. In our estimations, we employ “uniform count bin” histograms to estimate the marginal and joint pdf of the variables. Therefore, each datum of $V_{1}$ and $V_{2}$ variables is used to arrange the bins, in such a way that there are 15 data samples in the bins. Having estimated the pdf estimates, we substitute these in the marginal and joint entropies between the two action potentials in Equation (5). As a result of Monte Carlo simulations, we obtain the following behavior of MI, where the mean and the errorbars are ilustrated as a function of increasing noise levels:

In Figure 3a, we note that MI decreases as the noise level increases up to the vicinity of σ≅7. After this point, the MI values start rising by the increase at the noise level. This increase at MI versus σ means that the dependency value between the two neurons rises again. Although the noise effect acts as a corrupter until σ≅7, this effect gradually disappears and lets us infer the interaction between the neurons. Moreover, we can conclude that the 1-to-1 coupling case is less effected when compared to the 2-to-1 coupling case under noise levels up to σ≅7.

### 3.3. Dependency Analysis by Copulas

As introduced in the first section, we can use many copulas from two main families to infer the interactions between the neurons from observation data. To find the optimal copula, we suggest utilizing the Vuong–Clarke test [24,25,26] with 40 different copula types. Each copula candidate is scored by this test for 10 values of standard deviation (σ) pertaining to the additive Gaussian noise in Equations (16) and (17). We use Monte Carlo simulations to estimate the mean and variance of parameters. For example, using a single data set, where the voltage signals are coupled by *k* = 0.1 under a noise with σ = 7, the Clayton copula provides the highest score in the Vuong–Clarke test. Thus, it was chosen to model the dependency between the signals. By using the definition of this copula given in Equation (14), we can write the following likelihood function:

(18) $$l\left(\theta \right)=\sum_{k=1}^{N}\log C_{\theta }\left(u_{1},u_{2}\right)=\log\sum_{k=1}^{N}\frac{1}{\theta }\left[\frac{1}{u_{1,k}^{\theta }}+\frac{1}{u_{2,k}^{\theta }}-1\right]$$

In the next step, we use maximum likelihood estimation to find the optimal value of the parameter θ.

(19) $$\hat{\theta }=\underset{\theta }{\mathrm{argmax}}\left\{\frac{\partial l\left(\theta \right)}{\partial \theta }\right\}$$

**Figure 4:**
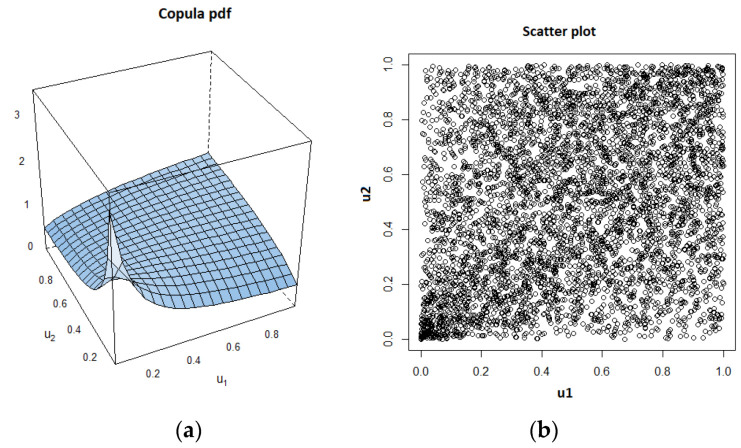
Estimated Clayton copula probability density function (pdf) and corresponding scatter plot. (**a**) Copula pdf; (**b**)Scatter plot.

As an example, we show the estimated copula pdf and the scatter plot, which were obtained using a data set for the case of *k* = 0.1 and σ = 10, in Figure 4.

Above, we notice the lower tail dependency between the variables. After determining the copula type and its parameter value, Kendall’s τ value is estimated by using the formula corresponding to the copula type, which is Equation (15), in case of Clayton copula.

**Figure 5:**
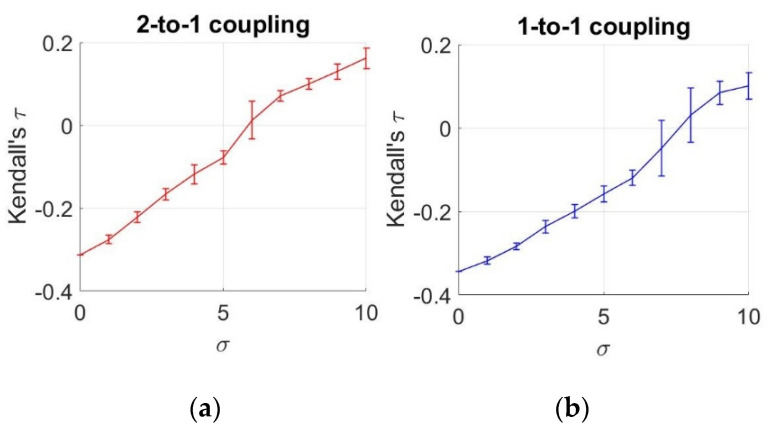
Estimates of Kendall’s τ as a function of noise levels. (**a**) 2-to-1 coupling case (*k* = 0.1); (**b**) 1-to-1 coupling case (*k* = 0.25).

To analyze the statistical dependencies between two neurons under different noise levels, we demonstrate the estimated mean and standard deviation of τ values from an ensemble of 10 members, as a function of increasing noise levels, as illustrated in Figure 5.

To obtain these values, we use an ensemble of 10 members for each σ value. Based on these results, we observe a negative relationship between the neurons under σ≅6 for *k* = 0.1 and under σ=7.5 for *k* = 0.25. After these values, we notice that the sign of dependency changes, meaning that an increase in one action potential is related to an increase in the other potential. However, until reaching these σ values, the sign of the relationship is found to be negative, which complies with the negative coupling in the HH equations of Equations (16) and (17). This negative interaction (increase in one variable causing decrase in the other) decreases until σ≅6 for *k* = 0.1 and under σ=7.5 for *k* = 0.25, and turns into a positive interaction after these points. This observation also verifies the results obtained by MI. Similar to this case, MI acts in two ways by decreasing until σ≅7 and by increasing after σ≅7, meaning that we observe the same positive relationship in the second part. 

### 3.4. Dependency Analysis by TE 

**Figure 6:**
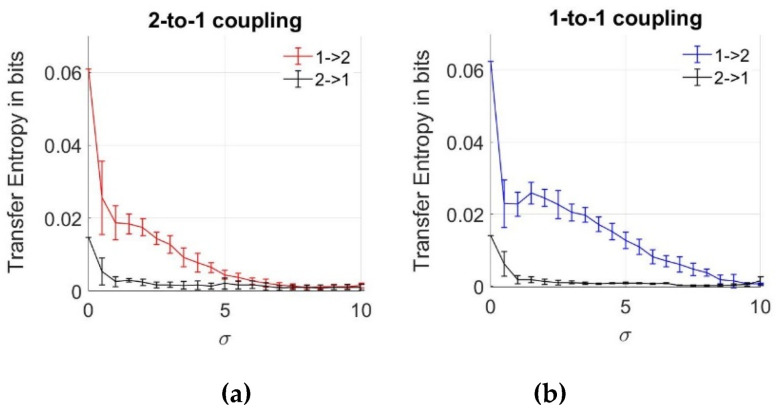
Transfer entropy estimates between two neurons as a function of noise standard deviation for two coupling values. (**a**) 2-to-1 coupling case (*k* = 0.1); (**b**) 1-to-1 coupling case (*k* = 0.25).

Previously, we emphasized that the MI and copulas are effective for the determination of the interaction between two coupled neurons despite the noise effect. However, we cannot obtain any information from data regarding the direction of the coupling. To analyze the information flow between the neurons, we estimate TE between the action potentials, as described in Section 2.2, using Equations (6) and (7), with increasing noise intensities of the noise components given in Equations (16) and (17). The mean and errorbar of each TE result is plotted in Figure 6 for both 2-to-1 and 1-to-1 coupled systems, using ensemble averaging over 10 data sets. As expected for the network without noise (σ = 0), transfer entropy value is the highest for both coupling coefficients. This verifies the changes caused in Neuron 2 by Neuron 1. For the low noise levels, we notice lower values of TE in the other direction from Neuron 2 to Neuron 1, which must be zero based on Equations (16) and (17). This observation is due to the bias effects of the numerical estimation algorithms. To see the effects of noise, we explore the TE between the neurons with increasing σ parameter. After σ = 10, neurons turn back to steady state and no more spiking pattern occurs. From Figure 6, we note that with the increasing noise intensity, values of TE from the first to the second neuron decrease, and their values are higher compared to those in the opposite direction, implying that the direction of information flow has been inferred correctly from data. Additionally, the effect of bias in TE estimation is observed to be decreasing with the increasing noise levels. As a result of the comparison between two different coupling coefficients, we notice that TE inference is still possible for σ > 7 up to σ = 10 for *k* = 0.25, while the inference is not possible (both zero in two directions from 1 to 2 and 2 to 1) above σ = 7 for a lower coupling magnitude where *k* = 0.1.

In Figure 6b, we notice that the average value of TE can also slightly increase, unlike the gradual decrease as a function of increasing noise levels. However, we notice that this increase at σ = 2 is within the errorbar, meaning that it is not statistically significant. In the supplementary files, we also show some ensemble members of TE and copula curves versus noise levels.

When we compare the results obtained by the MI and TE analyses, we notice that TE keeps decreasing even after σ = 7 and approaches zero, whereas MI and copulas can also infer the relationship that arises due to the effects of noise after σ = 7, which means that high level noise is effective in the inference of the dependency between the neurons, but not on the inference of the information flow. 

In the next section, we provide our conclusions and propose a generalization for high number of neuronal interactions.

## 4. Discussion and Conclusions

In this work, we propose a statistical approach to infer the statistical interactions between two coupled neurons from data under increasing noise levels. The neuronal action potential data are generated by the equations of the HH model, wherein the synaptic interactions are modeled by coupling coefficients. To apply the proposed statistical approaches, we generate data using this model in such a way that we can obtain 2-to-1 coupling and 1-to-1 coupling between the neurons under various levels of additive noise. We demonstrate the performance of each method on this data, for which we know the true connections, enabling us to assess our findings.

In order to infer these couplings despite noise, we attack the problem from two perspectives: First we analyze the symmetrical statistical dependencies by estimating the MI and copulas. Second, we examine the directional relationship between the neurons using TE. Based on the simulations, we notice a decrease followed by an increase in MI and copulas as the noise level increases. The initial decrease can be explained by the negative coupling from the first neuron to the second, as shown in the model. As the noise level increases, the dependency between two neurons decreases. However, as the noise level keeps rising, the MI and Kendall’s τ values start rising again, implying that the dependency between the two neurons increases. This relationship does not reflect the negative coupling effect and shows a positive dependency. This means that noise component causes this positive dependency as the effect of coupling diminishes. Thus, the results demonstrate two different interactions between the neurons due to the additive noise. 

In addition to these, we also note that the direction of information flow from the first neuron to the second can also be detected correctly for low noise levels, and the value of this quantity approaches to zero with increasing noise levels, which supports the previous results. This means that we are unable to infer the direction of the coupling between the two neurons for high noise range. In this range, the effects of noise suppress the effect of the coupling from the first neuron to the other. 

The results show that we can infer the statistical dependencies among coupled neurons using information theory’s quantities and copulas. This joint approach by three methods constitutes a promising tool that can help us infer the interactions between neurons in a larger network wherein multivariate versions of the proposed approach need to be investigated.

## Figures and Tables

**Table 1:** Initial values to solve the system and coupling constants for 1-1 and 2-1 coupling cases.

Initial values	*V*_1_ = *V*_2_ = −65; *m*_1_ = *m*_2_ = 0.05; *h*_1_ = *h*_2_ = 0.6; *n*_1_ = *n*_2_ = 0.317
1-to-1 coupling	*k* = 0.25
2-to-1 coupling	*k* = 0.1

## Supplementary Materials

The following are available online at https://www.mdpi.com/1099-4300/22/4/387/s1.

## Appendix A. Hodgkin–Huxley Model

In Hodgkin–Huxley model, conductance parameters are also voltage dependent: $g_{K}$ depends on four activation gates and defined as $g_{K}=\bar{g_{K}}n^{4}$ whereas $g_{Na}$ depends on three activation gates and one inactivation gate and modeled as $g_{Na}=\bar{g_{Na}}m^{3}h$. In the HH model, ionic currents are defined as follows:

(A1) $$I_{Na}=\bar{g_{Na}}m^{3}h\left(V-V_{Na}\right)\;,$$

(A2) $$I_{K}=\bar{g_{K}}n^{4}\left(V-V_{K}\right),$$

(A3) $$I_{L}=\bar{g_{L}}\left(V-V_{L}\right),$$

with Na^+^ activation variable *m* and inactivation variable *h*, and K^+^ activation variable *n*. Here $\left(\bar{.}\right)$ denotes maximal conductances. Activation and inactivation dynamics of the channels change according to the differential equations as shown below:

(A4) $$\frac{dm}{dt}=\frac{m_{\infty }\left(V\right)-m}{\tau_{m}\left(V\right)}\;,$$

(A5) $$\frac{dh}{dt}=\frac{h_{\infty }\left(V\right)-h}{\tau_{h}\left(V\right)}\;,$$

(A6) $$\frac{dn}{dt}=\frac{n_{\infty }\left(V\right)-n}{\tau_{n}\left(V\right)}\;.$$

The steady state activation and inactivation functions are defined as follows;

(A7) $$x_{\infty }\left(V\right)=\frac{\alpha_{x}\left(V\right)}{\alpha_{x}\left(V\right)+\beta_{x}\left(V\right)},\;x=m,h,n.$$

Time constants ($\tau_{x}$) represent the time that the channel needs to reach the equilibrium and are defined by the following equation:

(A8) $$\tau_{x}\left(V\right)=\frac{1}{\alpha_{x}\left(V\right)+\beta_{x}\left(V\right)},\;x=m,h,n$$

The transition rates $\alpha_{x}\;\mathrm{and}\;\beta_{x}$ together with the parameter values are given in Table A1.

**Table A1:** Transition rates and parameter values for the HH Model [42].

Transition rates (ms^−1^)	$\alpha_{m}$	0.1(40+V)/(1−exp(−(55+V)/10))
	$\beta_{m}$	4exp(−(65+V)/18)
	$\alpha_{h}$	0.07exp(−(65+V)/20)
	$\beta_{h}$	1/(1+exp(−(35+V)))
	$\alpha_{n}$	0.01(55+V)/(1−exp(−(10V+55)))
	$\beta_{n}$	0.125exp(−(V+65)/80)
Parameter Values	$C_{m}$	1 μF
	$I_{app}$	8 mA
	$g_{Na}$	120 μS
	$g_{K}$	36 μS
	$g_{L}$	0.3 μS
	$V_{Na}$	50 mV
	$V_{K}$	−77 mV
	$V_{L}$	−54.4 mV

## References

[1] Hodgkin A.L., Huxley A.F. (1952). A quantitative description of membrane current and its application to conduction and excitation in nerve. J. Physiol..

[2] Pandit S.V., Clark R.B., Giles W.R., Demir S.S. (2001). A Mathematical Model of Action Potential Heterogeneity in Adult Rat Left Ventricular Myocytes. Biophys. J..

[3] Bertram R., Sherman A. (2004). A calcium-based phantom bursting model for pancreatic islets. Bull. Math. Biol..

[4] Duncan P.J., Şengül S., Tabak J., Ruth P., Bertram R., Shipston M.J. (2015). Large conductance Ca^2+^-activated K^+^ (BK) channels promote secretagogue-induced transition from spiking to bursting in murine anterior pituitary corticotrophs. J. Physiol..

[5] Destexhe A. (2012). Neuronal Noise.

[6] Goldwyn J.H., Shea-Brown E. (2011). The what and where of adding channel noise to the Hodgkin-Huxley equations. PLoS Comput. Biol..

[7] Goldwyn J.H., Imennov N.S., Famulare M., Shea-Brown E. (2011). Stochastic differential equation models for ion channel noise in Hodgkin-Huxley neurons. Phys. Rev. E.

[8] Horikawa Y. (1991). Noise effects on spike propagation in the stochastic Hodgkin-Huxley models. Biol. Cybern..

[9] Moss F., Ward L.M., Sannita W.G. (2004). Stochastic resonance and sensory information processing: A tutorial and review of application. Clin. Neurophysiol..

[10] White J.A., Rubinstein J.T., Kay A.R. (2000). Channel noise in neurons. Trends Neurosci..

[11] Ermentrout G.B., Galán R.F., Urban N.N. (2008). Reliability, synchrony and noise. Trends Neurosci..

[12] Faisal A.A., Selen L.P., Wolpert D.M. (2008). Noise in the nervous system. Nat. Rev. Neurosci..

[13] Lee K.-E., Lopes M.A., Mendes J.F.F., Goltsev A.V. (2014). Critical phenomena and noise-induced phase transitions in neuronal networks. Phys. Rev. E.

[14] Lindner B. (2004). Effects of noise in excitable systems. Phys. Rep..

[15] Brown E.N., Kass R.E., Mitra P. (2004). PMultiple neural spike train data analysis: State-of-the-art and future challenges. Nat. Neurosci..

[16] Cohen M.R., Kohn A. (2011). Measuring and interpreting neuronal correlations. Nat. Neurosci..

[17] Yamada S., Nakashima M., Matsumoto K., Shiono S. (1993). Information theoretic analysis of action potential trains. Biol. Cybern..

[18] Yamada S., Matsumoto K., Nakashima M., Shiono S. (1996). Information theoretic analysis of action potential trains II. Analysis of correlation among n neurons to deduce connection structure. J. Neurosci. Methods.

[19] Wibral M., Vicente R., Lizier J.T. (2014). Directed Information Measures in Neuroscience.

[20] Li Z., Li X. (2013). Estimating Temporal Causal Interaction between Spike Trains with Permutation and Transfer Entropy. PLoS ONE.

[21] Ito S., Hansen M.E., Heiland R., Lumsdaine A., Litke A.M., Beggs J.M. (2011). Extending Transfer Entropy Improves Identification of Effective Connectivity in a Spiking Cortical Network Model. PLoS ONE.

[22] Walker B.L., Newhall K.A. (2018). Inferring information flow in spike-train data sets using a trial-shuffle method. PLoS ONE.

[23] Nelsen R.B. (2006). An Introduction to Copulas.

[24] Belgorodski N. (2010). Selecting Pair-Copula Families for Regular Vines with Application to the Multivariate Analysis of European Stock Market Indices. Diplomarbeit.

[25] Clarke K.A. (2007). A Simple Distribution-Free Test for Nonnested Model Selection. Political Anal..

[26] Vuong Q.H. (1989). Ratio tests for model selection and non-nested hypotheses. Econometrica.

[27] Schreiber T. (2000). Measuring information transfer. Phys. Rev. Lett..

[28] Gencaga D. (2018). Transfer Entropy (Entropy Special Issue Reprint).

[29] Gencaga D., Knuth K.H., Rossow W.B. (2015). A Recipe for the Estimation of Information Flow in a Dynamical System. Entropy.

[30] Knuth K.H. (2006). Optimal data-based binning for histograms. arXiv.

[31] Scott D.W. (2015). Multivariate Density Estimation: Theory, Practice, and Visualization.

[32] Darbellay G.A., Vajda I. (1999). Estimation of the information by an adaptive partitioning of the observation space. IEEE Trans. Inf. Theory.

[33] Timme N.M., Lapish C.C. (2018). A tutorial for information theory in neuroscience. eNeuro.

[34] Brechmann E.C., Schepsmeier U. (2013). Modeling Dependence with C- and D-Vine Copulas: The R Package CDVine. J. Stat. Softw..

[35] Dhanya E., Sunitha R., Pradhan N., Sreedevi A. Modelling and Implementation of Two Coupled Hodgkin-Huxley Neuron Model. Proceedings of the 2015 International Conference on Computing and Network Communications.

[36] Ao X., Hanggi P., Schmid G. (2013). In-phase and anti-phase synchronization in noisy Hodgkin–Huxley neurons. Math. Biosci..

[37] Cramer H. (1946). Mathematical Methods in Statistics.

[38] Rudolph M., Destexhe A. (2001). Correlation Detection and Resonance in Neural Systems with Distributed Noise Sources. Phys. Rev. Lett..

[39] Paninski L. (2003). Estimation of entropy and mutual information. Neural Comput..

[40] Verdú S. (2019). Empirical Estimation of Information Measures: A Literature Guide. Entropy.

[41] Ermentrout G. (2002). Simulating, Analyzing, and Animating Dynamical Systems.

[42] Şengül S., Clewley R., Bertram R., Tabak J. (2014). Determining the contributions of divisive and subtractive feedback in the Hodgkin-Huxley model. J. Comput. Neurosci..

