# Supplementary material for: Statistical Approaches for the Analysis of Dependency Among Neurons Under Noise
Source: Entropy (Basel). 2020 Mar 28;22(4):387. doi: 10.3390/e22040387 (PMC7516863; doi:10.3390/e22040387)
Supplement: Supplementary file 1 [file entropy-22-00387-s001.pdf]

**Supplementary Materials:**

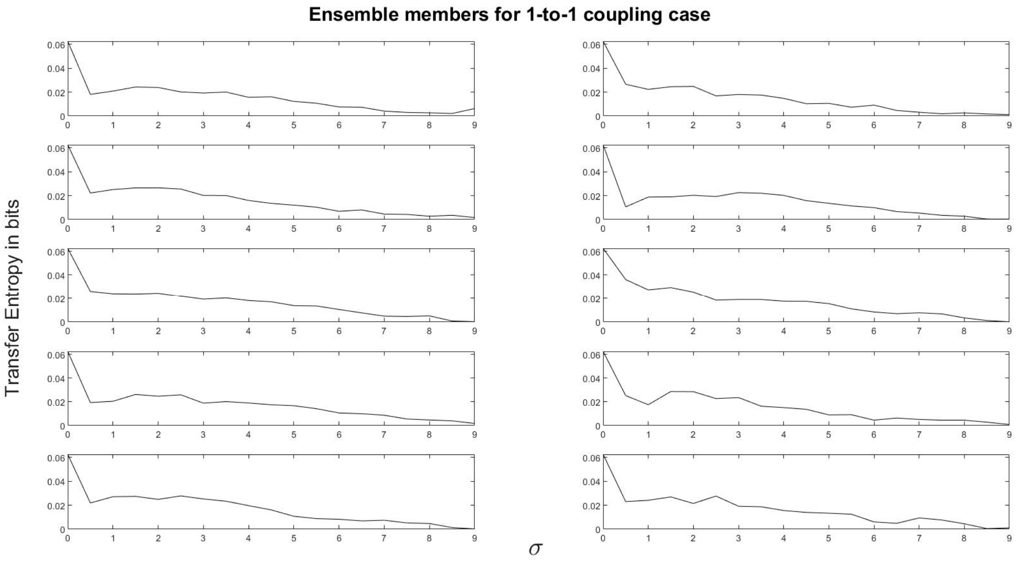


Figure S1. Transfer Entropy values versus noise standard deviations for each member in the ensemble

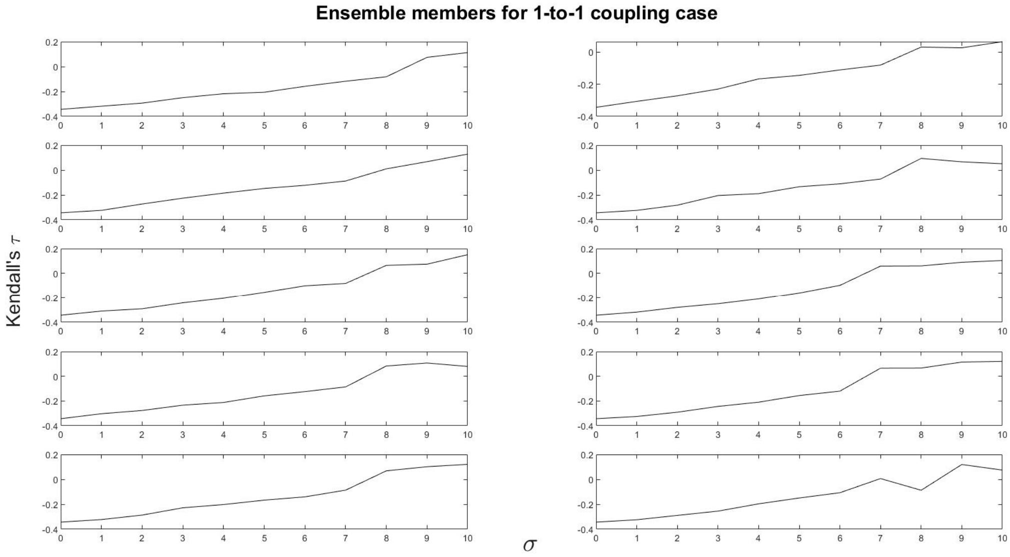


Figure S2. Kendall’s τ values versus noise standard deviations for each member in the ensemble

**XPPAUT code used in the generation of coupled action potential data under noise by simulating Equations (16) and (17)**

```
#  Hodgkin huxley equations
v1'=(i1-gna1*m1^3*h1*(v1-vna)-gk1*n1^4*(v1-vk)-gl1*(v1-vl)-inoise)/c
n1'=an(v1)*(1-n1)-bn(v1)*n1
m1'=am(v1)*(1-m1)-bm(v1)*m1
h1'=ah(v1)*(1-h1)-bh(v1)*h1
v2'=(-gna2*m2^3*h2*(v2-vna)-gk2*n2^4*(v2-vk)-gl2*(v2-vl)-k2*(v1-v2)-inoise)/c
n2'=an(v2)*(1-n2)-bn(v2)*n2
m2'=am(v2)*(1-m2)-bm(v2)*m2
h2'=ah(v2)*(1-h2)-bh(v2)*h2

an(v)=.01*(v+55)/(1-exp(-(v+55)/10))
bn(v)=.125*exp(-(v+65)/80)
am(v)=.1*(v+40)/(1-exp(-(v+40)/10))
bm(v)=4*exp(-(v+65)/18)
ah(v)=.07*exp(-(v+65)/20)
bh(v)=1/(1+exp(-(v+35)/10))

#an(v)=.01*(10-v)/(exp((10-v)/10)-1)
#bn(v)=.125*exp(-(v/80))
#am(v)=.1*(25-v)/(exp((25-v)/10)-1)
#bm(v)=4*exp(-(v)/18)
#ah(v)=.07*exp(-(v)/20)
#bh(v)=1/(1+exp((-v+30)/10))

wiener w
inoise = sigma*w

init v1=-65  m1=.05  h1=0.6  n1=.317
init v2=-65  m2=.05  h2=0.6  n2=.317
par i1=8, c=1, k1=0, k2=0.25
par vna=50  vk=-77  vl=-54.4
par gna1=120  gk1=36  gl1=0.3
par gna2=120  gk2=36  gl2=0.3
par sigma=0.5


# track the stimulus
@ nplot=2, yp1=v1, yp2=v2
@ bound=10000000, total=2000
done
```
